# Immune Responses to IAV Infection and the Roles of L-Selectin and ADAM17 in Lymphocyte Homing

**DOI:** 10.3390/pathogens11020150

**Published:** 2022-01-25

**Authors:** Sophie G. Reed, Ann Ager

**Affiliations:** Division of Infection and Immunity, School of Medicine, Cardiff University, Cardiff CF14 4XN, UK

**Keywords:** L-selectin, ADAM17, influenza A virus, T cells, leukocyte homing

## Abstract

Influenza A virus (IAV) infection is a global public health burden causing up to 650,000 deaths per year. Yearly vaccination programmes and anti-viral drugs currently have limited benefits; therefore, research into IAV is fundamental. Leukocyte trafficking is a crucial process which orchestrates the immune response to infection to protect the host. It involves several homing molecules and receptors on both blood vessels and leukocytes. A key mediator of this process is the transmembrane glycoprotein L-selectin, which binds to vascular addressins on blood vessel endothelial cells. L-selectin classically mediates homing of naïve and central memory lymphocytes to lymph nodes via high endothelial venules (HEVs). Recent studies have found that L-selectin is essential for homing of activated CD8^+^ T cells to influenza-infected lungs and reduction in virus load. A disintegrin and metalloproteinase 17 (ADAM17) is the primary regulator of cell surface levels of L-selectin. Understanding the mechanisms that regulate these two proteins are central to comprehending recruitment of T cells to sites of IAV infection. This review summarises the immune response to IAV infection in humans and mice and discusses the roles of L-selectin and ADAM17 in T lymphocyte homing during IAV infection.

## 1. Influenza Virus

Viruses are small intracellular parasites which can infect animals, plants or microorganisms. Many viruses, including influenza A virus (IAV), are unable to self-replicate and require a host to survive by inserting their genome into the host cells’ replication machinery to create new virions. Influenza viruses are of the orthomyxovirus family and can be categorised into types A, B, C and D. Influenza D primarily infects cattle, whilst influenza C is known to cause only mild illness in humans [1]. Types A and B cause seasonal epidemics each year; however, only the A subtype has resulted in worldwide pandemics to date. Influenza virus is a significant health burden throughout the world, causing up to 650,000 deaths per year [1]. Typical influenza virus infection presents with respiratory symptoms such as a cough, sore throat and rhinitis as well as fever, headache and myalgia. In severe cases, which mainly occur in the elderly and those with pre-existing conditions, viral or secondary bacterial pneumonia can occur leading to sepsis and organ failure [1]. Annual vaccinations are recommended for the public but predicting circulating strains can be highly challenging with such a dynamic virus. IAV is known to cause higher mortality than other influenzas, and so it is therefore more widely researched.

Highly pathogenic viruses have the ability to cause worldwide pandemics such as Spanish Influenza (H1N1) in 1918, Swine Influenza (H1N1) in 2009 and the most recent ongoing SARS-CoV-2 (COVID-19) pandemic. Due to the increasing possibility of future pandemics for which there are no vaccines or anti-viral drugs available, investigating immune responses and therapeutics that target the immune system are both fundamental, to limit virus-induced pathology and boost immune-mediated destruction of virus-infected cells. 

Leukocyte homing encompasses the migration of immune cells throughout the body. Different types of leukocyte need to traffic to specific tissues at specific timepoints to maintain homeostasis, eliminate infections and kill cancers. Leukocyte homing is orchestrated by multiple sets of receptors and ligands on both leukocytes and endothelial cells. Understanding the molecular basis of leukocyte homing will be key for manipulating the immune response therapeutically to either elicit cytotoxic responses to an infection, or to limit excessive inflammation which causes tissue damage. One of the most researched leukocyte homing molecules is L-selectin (CD62L), which has been directly linked to improved CD8^+^ T cell homing and virus clearance in a mouse model of IAV infection [2]. L-selectin, along with its physiological regulator, A disintegrin and metalloproteinase 17 (ADAM17), are the focus of this review on leukocyte homing in the context of IAV infection.

## 2. Tissue Tropism

Influenza virus enters the host through the respiratory tract. The haemagglutinin (HA) glycoprotein on the virion surface has receptor binding and membrane fusion capabilities to allow entry into host cells. The HA receptor binding domain binds sialylated glycoconjugates on the host cell surface. α2,3 or α2,6 sialic acid (N-acetyl neuraminic acid) linkages are preferentially targeted by HA in humans [3].

Epithelial cells (pneumocytes in the alveoli) are one of the first cells contacted by IAV within the respiratory tract, making them highly susceptible to initial infection. In animal and human lung tissue sections IAV strains H1N1 and H3N2 demonstrated preferential binding to type I pneumocytes, while H5N1, H5N9, and H6N1 bound to type II pneumocytes [4]. Ex vivo infection of human lung explants showed association of viral antigen with type II pneumocytes for H5N1, H1N1 and H3N2 strains [5].

Alveolar macrophages (AMs) are the resident population of phagocytes present in the lungs, which have roles in homeostasis and defense against pathogens. Evidence from ex vivo studies on primary human cells suggests that AMs can also become directly infected by IAV, but to a much lesser extent than epithelial cells [6,7]. However, mouse AMs are more susceptible to direct infection with up to 90% of primary murine AMs becoming infected, dependent on IAV strain [8]. As well as AMs, a range of immune cells such as monocytes, dendritic cells, neutrophils, natural killer (NK) cells, eosinophils and B cells are found to be directly infected by IAV in humans [9,10,11,12,13], and mice [14,15,16]. Some studies suggest this can be abortive infection, whereby the cells infected do not produce functional IAV virions [13,15]. Other studies suggest these cells may be ‘productively infected’ and able to migrate and disseminate the virus to other organs or tissues [11]. This could be a mode of infection for blood vessel endothelial cells; however, there is limited data available to suggest endothelial cells become infected in mammals [17]. H5N1 viral RNAs have been found in peripheral blood and rectum of patients who died of the virus [18], as well as detectable virus in multiple organs including the brain, spleen and intestine of H5N1 infected ferrets [19]. Further complications such as myocarditis, stroke, encephalitis, acute kidney injury (AKI) and rhabdomyolysis among others, have been documented in severe influenza cases [20]. However, it is currently unclear whether these complications are a direct effect of virus on the organs themselves, or whether it is a secondary effect caused by a heightened immune response [20]. 

## 3. Mouse Models for Influenza Virus Research

In early studies, pigs, ferrets and mice were found to be excellent models for studying influenza virus in vivo [21]. Due to accessibility, cost, husbandry requirements, ease of handling, experimental numbers and availability of species-specific reagents, mice have been adopted as a key model for in vivo influenza virus research. Immune responses are comparable between humans and mice, with strong inflammatory responses being the trigger for disease-associated lung pathology [22]. Mice exhibit similar histological and pathological changes in the respiratory tract to humans with IAV [21]; however, clinical signs of influenza vary greatly between species [23]. Key symptoms in humans include fever, myalgia, headache and respiratory symptoms such as cough or congestion, whereas in mice anorexia, hypothermia and lethargy are prevalent [23]. A further notable difference between human and mouse IAV infection is that influenza presents itself primarily as an upper respiratory tract infection in mild to moderate disease in humans, whereas in mice it predominantly affects the lower respiratory tract [4,23]. 

Mouse-adapted strains of influenza are required for effective infection and replication in mice, due to lack of α2,6 sialic acid receptors on mouse lung epithelial cells [24]. The most commonly used strains of IAV are H1N1 strains A/Puerto Rico/8/1934 (PR8), A/WSN/1933 (WSN) or H3N2 strain A/X-31 (X31). However, these strains are not circulating human isolates and therefore may not be clinically relevant. Pathogenicity of IAVs and susceptibility to infection also varies greatly depending on mouse strain [25].

Recent investigations into the use of specific pathogen-free (SPF) mice for biomedical research has questioned the translational potential to human disease. Mice which live in the wild were found to mirror the human immune system much more closely than lab-bred strains of mice [26]. Lab-bred mice co-housed with wild mice were found to have transcriptional signatures similar to humans when challenged with influenza vaccination, compared to lab-bred mice [27]. This suggests SPF-housed in-bred strains of mice used in research may not be beneficial for effectively representing the ‘dirty’ way in which humans live, undergoing constant challenge to the immune system. 

On the contrary, a recent study concluded that laboratory mice are an excellent model for discovering much needed genetic biomarkers for influenza virus infection [28]. Additionally, mice have been used for preliminary in vivo studies of multiple anti-viral drugs, which are now approved in humans such as amantadine, rimantadine, Tamiflu^®^, Relenza^®^ and peramivir [29,30,31,32,33]. These findings suggest mouse models are pivotal in the discovery of therapeutics for controlling IAV infection in humans.

## 4. Innate Immune Responses to IAV Infection and Immunopathology

Both the innate and adaptive immune responses are required to eliminate IAV infection. The first barriers to infection are physical defenses such as mucous lining the respiratory tract, which contains glycosylated mucins to limit virus binding to respiratory epithelium [34,35]. Once physical barriers are breached, the virus will infect the airway epithelium, kill the infected cells and trigger the innate immune response. The immune response is a finely tuned mechanism which functions to elicit inflammation and cytotoxicity to kill an invading pathogen, whilst releasing anti-inflammatory factors to limit host tissue damage. This response can sometimes become imbalanced leading to extensive pathology to the host, or inability to control infection. Due to the clear roles of innate immunity in both clearing IAV infection and causing pathology, manipulating leukocyte homing in vivo may be a difficult challenge. The implicated immune cells in IAV infection and their roles in immunoprotection and immunopathology are summarised in Table 1.

## 5. Adaptive Immune Responses to IAV Infection

### 5.1. Roles of the Draining Lymph Nodes

The mediastinal lymph nodes are the lung-draining lymph nodes in both humans and mice. They function to harbour immune cells for immunosurveillance, antigen priming and adaptive immune cell activation to limit infection spread. The mediastinal lymph node in mice increases in both size and cellularity within 48 h of IAV infection, due to rapid activation of the immune response [55]. Within the upper airways, cervical lymph nodes and nasal-associated lymphoid tissue (NALT) are the draining lymph nodes.

**Figure 1:**
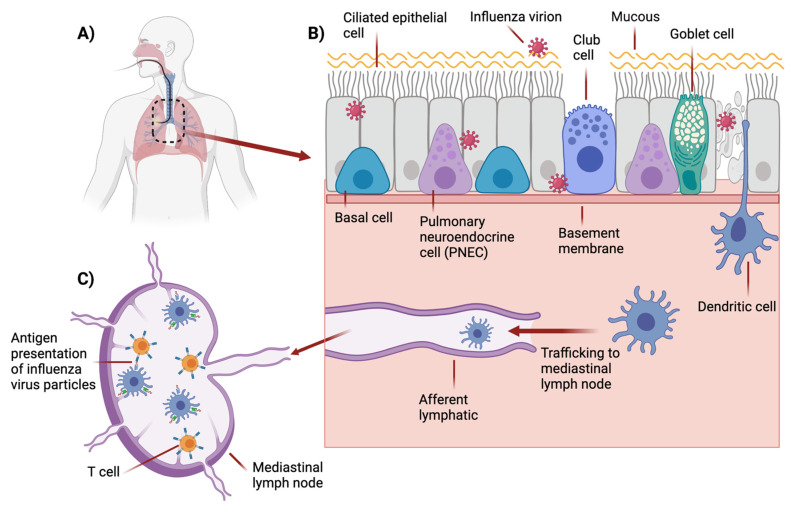
Influenza infection of airway epithelium leads to activation of the innate, then adaptive immune response in the mediastinal lymph node. (**A**) The human respiratory system with the trachea and bronchi circled. (**B**) Human airway epithelial layer consisting of ciliated epithelial cells, club cells, goblet cells, basal cells and pulmonary neuroendocrine cells (PNEC) with basement membrane. Influenza virions infect epithelial cells, resulting in cell death. Dendritic cells (DCs) take up dead or live virus particles and traffic in afferent lymphatics to mediastinal lymph nodes. (**C**) Within mediastinal lymph node, DCs present antigen to T cell receptors (TCRs) on T cells via major histocompatibility complex I or II (MHC-I or MHC-II), leading to T cell activation and differentiation, inducing the adaptive arm of the immune system.

Activation of the adaptive immune response to IAV relies on antigen-presenting cells (APCs) such as CD11b^low/neg^CD103^+^ dendritic cells (DCs) to survey the lungs, phagocytose virus particles and upregulate chemokine receptor 7 (CCR7) [56]. They then enter afferent lymphatics via CCR7 interaction with chemokine ligands 19 and 21 (CCL19, CCL21) and are transported to the draining lymph nodes [57]. Within the draining lymph nodes, naïve CD4^+^ and CD8^+^ T cells recruited from the bloodsteam via high endothelial venules (HEVs), circulate in the paracortex (T cell zone) where they survey peptide-MHC (major histocompatibility complex) on DCs. DCs carrying IAV peptides will present the epitope on MHC-I or MHC-II, and an antigen-specific T cell receptor (TCR) on a T cell will bind the MHC, along with its corresponding CD8 or CD4 co-receptor. Recognition of foreign peptides through this process will initiate the adaptive arm of the immune system (Figure 1).

### 5.2. Roles of Lymphocytes

Following activation and differentiation in lymph nodes, cytokine secreting CD8^+^ cytotoxic T lymphocytes (CTLs) downregulate CCR7 which recognises CCL19 and CCL21 expressed within HEV and paracortex, as well as downregulating L-selectin [56,57,58]. This is accompanied by upregulation of sphingosine-1-phosphate receptor (S1PR) which senses high levels of its ligand of sphingosine-1-phosphate (S1P) in lymph, allowing egress from the lymph node via efferent lymphatics and release into the bloodstream [59]. CTLs also upregulate various inflammation-associated chemokine receptors (e.g., CXCR3, CCR5) allowing subsequent recruitment to the site of infection to carry out cytotoxic responses [60].

CTLs limit viral replication by directly killing IAV-infected cells via cytotoxic granules and controlled apoptosis; making them one of the key components of a successful immune response against influenza virus. Upon recognition of an infected cell, CTLs will release perforin to create pores in the cell membrane, allowing granzymes A and B, also released by CTLs, to induce cell death. Expression of tumour necrosis factor (TNF)-related apoptosis inducing ligand (TRAIL) and Fas ligand (FasL) can also activate death receptors within infected cells, resulting in programmed cell death [61]. These ‘killer’ mechanisms protect against further viral infection and replication within the host. 

CD8^+^ T cells have been shown as vital in murine influenza virus infection, as mice lacking CD8^+^ T cells have a much higher mortality rate against H1N1 IAV than wild-type mice [62]. However, there is conflicting evidence for the role of CD8^+^ T cells in IAV infection, as CTLs produce the anti-inflammatory cytokine interleukin 10 (IL-10) to limit excessive inflammation and lung injury in murine IAV infection, and have been found to be protective in mild infection, yet can be pathogenic in severe infection [63,64]. Complete depletion of all lymphocytes in mice (Rag^−/−^) is lethal in IAV infection, suggesting that the innate immune system alone is insufficient to clear influenza virus infection [65].

Naïve CD4^+^ T cells are also activated within the draining lymph nodes in IAV infection via TCR binding to MHC-II bound IAV peptides on APCs. CD4^+^ T cells are classed as ‘T- helper cells’ (Th) and differentiate into a Th1 phenotype during viral infection driven by the transcription factor T-bet [66]. This initiates release of interferon gamma (IFNγ), TNFα and IL-2 [66]. Primary roles of CD4^+^ T cells during IAV infection include regulating CD8^+^ T cell responses and stimulating B cell responses [67,68]. A subset of CD4^+^ T cells will migrate away from the mediastinal lymph node to carry out effector functions in the lungs, whilst a subset of CXCR5^+^ CD4^+^ T cells deemed ‘T follicular helper cells’ (T_FH_), will migrate to the B cell zone to engage antigen presenting B cells. This results in antibody production, plasma cell and memory B cell generation [68]. Antigen-specific CD4^+^ T cells are detectable in the lungs of IAV infected mice 7 days post-infection, peaking at 10 days [69]. In the lungs of IAV infected mice, the predominant subset of CD4^+^ T cells are Th1 cells; however, T-regulatory cells (T_regs_) and T_FH_ cells are present in fewer numbers [69]. CD4^+^ T_regs_ are found to have important roles in dampening inflammatory responses during IAV infection, by reducing monocyte and macrophage accumulation [70].

B cells are another arm of the adaptive immune response, primarily involved in antibody production against foreign pathogens. Neutralising antibodies bind directly to IAV virions via HA epitopes, to limit host cell binding and infectivity, rendering the virus inactive. Non-neutralising antibodies also have indirect immunoprotective roles such as facilitating memory CD8^+^ T cell expansion, complement activation, antibody-dependent cellular cytotoxicity (ADCC) and phagocytosis of infected cells [71,72]. B cells are key in generating immune protection from IAV vaccines, as memory CD8^+^ T cells alone are found not to protect mice against secondary heterosubtypic IAV challenge [71]. As early as 48 h post-infection in mice, extrafollicular plasmablasts secrete antibodies IgG, IgM and IgA, and in humans plasmablast levels peak in the blood on day 7 [55,73]. Later in infection, germinal centre B cells turn into long-lived plasma cells and memory B cells which may protect against subsequent IAV infections [72,74]. 

## 6. The Role of Lymphocyte Homing in Adaptive Immune Responses to IAV

As described, leukocytes function to protect the host from virus infections. To detect and eradicate these threats, both innate and adaptive immune cells must travel to sites of infection, as well as to draining lymph nodes or other lymphoid organs, in a tightly controlled manner. Leukocyte homing is controlled by different types of receptors on the leukocyte surface which bind to their respective ligands on blood vessel or HEV endothelium. This enables leukocytes to stably adhere to blood vessels and transmigrate the vessel wall to enter tissues. Leukocyte–endothelial cell interactions are stringently controlled to ensure leukocytes migrate to where they are needed, such as to the lungs and mediastinal lymph nodes in influenza infection, without causing excessive build-up of leukocytes and causing lung pathology. 

Most in vivo research into leukocyte homing is carried out in mice, whilst the majority of in vitro research is carried out using cultured human endothelial cells such as human umbilical vein endothelial cells (HUVECs). Some, but not all homing receptor-ligand interactions are conserved across species and collectively, this research can be used to predict mechanisms utilised by leukocytes for trafficking in humans. The next sections will describe the general principles underlying leukocyte homing and summarise the receptors and ligands that control homing of T cells to lymph nodes and virus-infected lungs.

### 6.1. The Multistep Adhesion Cascade of Leukocyte Homing

The multi-step adhesion cascade describes the sequence of adhesive interactions that regulate the recruitment of leukocytes from the bloodstream into tissues (also known as homing) and involves selectins, integrins and chemokines. The first step involves leukocyte tethering to the endothelium, which leads to slow rolling of the cell. Leukocytes then undergo arrest, and finally extravasation across the endothelial cell lining and vascular basement membrane to enter tissues [75].

**Figure 2:**
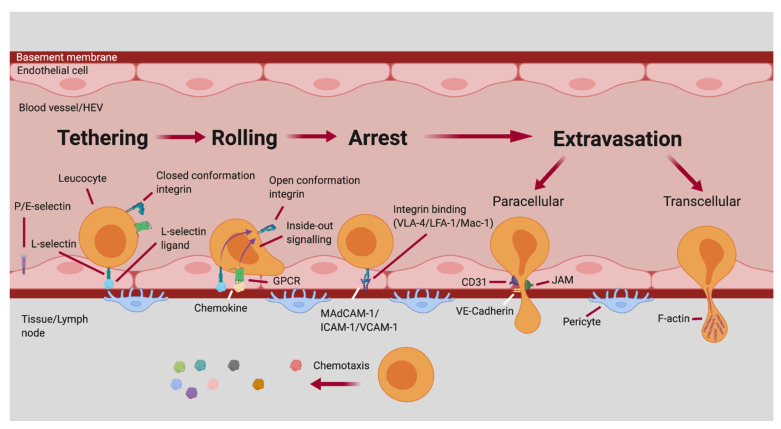
Leukocyte adhesion cascade within blood vessel/HEV. Leukocytes are recruited to lymph nodes or infected/inflamed tissues from high endothelial venules (HEVs) or inflamed blood vessels in a series of 4 major steps. Leukocytes tether to endothelium via the transmembrane receptors P-, E- or L-selectin which bind vascular addressins such as P-selectin glycoprotein 1 (PSGL-1), CD44 or peripheral lymph node addressin (PNAd). Leukocytes then slowly roll across the endothelium and come into contact with chemokines which bind G-protein coupled receptors (GPCRs). These events result in an inside-out signalling cascade stabilising integrin expression on the leukocyte such as leukocyte function-associated antigen 1 (LFA-1), very late antigen-4 (VLA-4) and macrophage antigen-1 (Mac-1) which bind mucosal vascular addressin cell-adhesion molecule 1 (MAdCAM-1), and immunoglobulin superfamily members intercellular cell-adhesion molecule-1 (ICAM-1) and vascular cell-adhesion molecule-1 (VCAM-1) on the endothelial cell surface. This causes arrest of the leukocyte onto the endothelium, ready for the final step of extravasation into the lymph node/tissue via paracellular or transcellular transmigration. Paracellular migration occurs via leukocyte interaction with junction adhesion molecules and release of VE-cadherin, which allows the leukocyte to pass between two endothelial cells. Transcellular migration occurs via F-actin rich podosomes on leukocytes passing through the cytoplasm of a single endothelial cell.

Selectins are type I transmembrane glycoproteins; P-selectin is found on platelets and endothelium, E-selectin is found only on endothelium and L-selectin is only on leukocytes. E- and P-selectins bind sialyl-lewis X (sLe^x^) epitopes on scaffold proteins such as P-selectin glycoprotein ligand 1 (PSGL-1) and CD44, whereas L-selectin binds to sulphated sLe^x^ on proteins such as CD34, podocalyxin and glycosylation-dependent cell adhesion molecule-1 (GlyCAM-1). These interactions initiate the tethering and rolling of cells on vasculature. Chemokine binding to glycosaminoglycans on activated endothelium further activates the slow-rolling leukocyte via G-protein coupled receptor (GPCR) activation of leukocytes. For example, CCR7 on leukocytes binding to CCL21 or CCL19 on HEV endothelium is key in T cell homing to lymph nodes, whereas CXCR5 binding to CXCL13 is key in B cell homing to lymph nodes. Chemokine and selectin binding also trigger inside-out signalling, which results in high-affinity, stabilised integrin expression. Integrins are cell surface receptors such as α4β7, leukocyte function-associated antigen 1 (LFA-1), very late antigen-4 (VLA-4) and macrophage antigen-1 (Mac-1), which bind molecules such as mucosal vascular addressin cell-adhesion molecule 1 (MAdCAM-1), and immunoglobulin superfamily members intercellular cell-adhesion molecule-1 (ICAM-1) and vascular cell-adhesion molecule-1 (VCAM-1). Once rolling cells are arrested by integrins, cells will begin to migrate through the endothelium, basement membrane and pericytes to reach their destination via one of two routes; paracellular or transcellular transmigration. Leukocyte engagement of junction adhesion molecules (JAMs); JAM-A, JAM-B, JAM-C, CD31 or CD99 will dissociate the binding of 2 adjacent endothelial cells. Vascular-endothelial cadherin (VE-cadherin) is also required to be released from the endothelium for this dissociation; these processes result in paracellular migration of the leukocyte between endothelial cells. Transmigration across a single endothelial cell, known as transcellular migration, can also occur via F-actin containing podosomes on the leukocyte, which allow transit through the endothelial cell cytoplasm. Migration through the basement membrane occurs at areas of low-density matrix proteins, which co-localise with gaps between adjacent pericytes, to allow the cell to reach the site of infection or lymph node via chemotaxis (Figure 2) [75,76,77].

ADAM17 is a transmembrane enzyme responsible for the cleavage of over 80 cell surface molecules [78]. Early in vitro studies with hydroxamate metalloproteinase inhibitors such as Ro 31-9790 and endogenous ADAM protease inhibitors such as tissue inhibitor of metalloproteinases-3 (TIMP-3) highlighted a potential role for ADAM17 in leukocyte homing [79]. ADAM17-dependent ectodomain cleavage controls the expression of several cell trafficking proteins such as L-selectin on leukocytes and VCAM-1 on endothelial cells. Studies using genetically modified leukocytes suggested roles for ADAM17 and shedding of L-selectin in innate immune cell recruitment in vivo [80] and for ADAM17 and L-selectin shedding in monocyte transendothelial migration in vitro [81]. The roles of L-selectin shedding and ADAM17 in lymphocyte homing will be covered in more detail later in this review.

### 6.2. T Lymphocyte Homing to Lymph Nodes and Lungs during IAV Infection

Each organ or tissue requires the expression of a unique set of homing molecules on vasculature and leukocytes to allow for optimal cell recruitment. Homing pathways vary depending on whether the secondary lymphoid organ is peripheral or mucosal. The majority of studies of in vivo peripheral and mucosal lymphocyte homing pathways have been carried out on naïve mice; physiological changes to cell trafficking during infection and inflammation are comparatively less well understood. However, there are currently no studies identifying T cell homing to the mediastinal lymph node in naïve mice, nor during infection. Due to the mediastinal lymph node being mucosal, it may have similar mechanisms to the mesenteric lymph node. Therefore, the trafficking mechanisms of T lymphocytes to and from the mediastinal lymph node and lungs during IAV infection is still to be elucidated (Table 2).

The lung alveoli contain the largest vascular bed in the body. This is important for both gas exchange and leukocyte recruitment due to the contact this organ has with the outside environment, rendering the lungs vulnerable to infection. There are three major vascular networks associated with respiratory tract infections; HEVs in mediastinal lymph nodes; tracheal and bronchial post-capillary vessels; and alveolar capillaries, all of which must adapt greatly in pulmonary infection to recruit leukocytes [96].

Tracheal and bronchial post-capillary vessels in the lungs constitutively express the integrin ligands VCAM-1 and ICAM-2 and various chemokines to allow for transient entry of leukocytes for surveillance. This alters during influenza virus infection whereby P- and E-selectin and inflammatory cytokines such as CXCL1 and CCL5 are upregulated on activated endothelium to promote recruitment of innate and adaptive immune cells. The pro-inflammatory chemokines and cytokines released, which cause these endothelial changes, are expressed by infected epithelial cells and AMs. During IAV infection, leukocytes upregulate cell surface expression of chemokine receptors such as CCR1, CCR3, CCR5, CXCR3 and integrins VLA-4, LFA-1 and Mac-1, which bind to ligands expressed on the activated endothelium [96].

Alveolar capillaries use slightly different cell homing mechanisms to post-capillary venules; selectins are not required for cell adhesion and rolling along this endothelium; however, similar chemokine receptors and integrins are expressed on cells trafficking through alveolar capillaries, with the exception of VLA-4 [96]. These differences are attributed to the narrow diameter of these capillaries, which induces a slower leukocyte motility as the majority of segments are narrower than the size of leukocytes trafficking through [96,97].

## 7. L-Selectin

**Figure 3:**
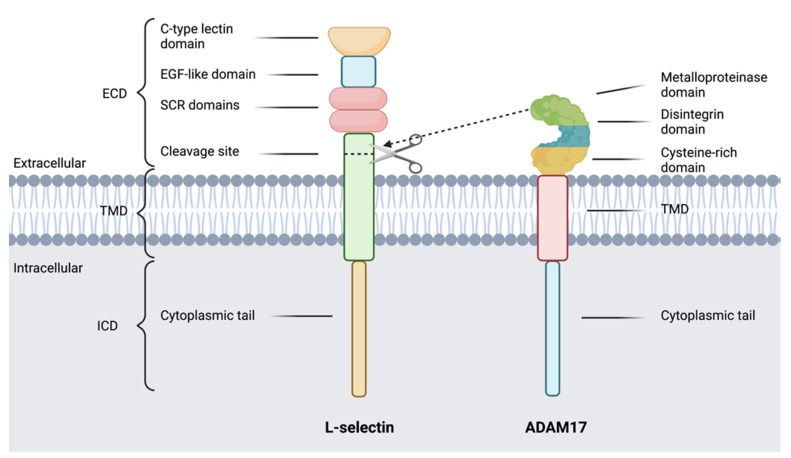
The structure of transmembrane glycoprotein L-selectin and enzyme ADAM17. L-selectin (CD62L) is comprised of an intracellular domain (ICD) at the C-terminus which consists of the 17 amino acid cytoplasmic tail, a transmembrane domain (TMD), which spans the cell membrane and an extracellular domain (ECD) involving the C-type lectin domain at the N-terminus, the epidermal growth factor (EGF)-like domain, two short consensus repeat domains and the cleavage site. L-selectin is proteolytically shed by A disintegrin and metalloproteinase 17 (ADAM17), which contains an ECD consisting of the metalloproteinase domain, disintegrin domain and cysteine-rich domain, which contains the hypervariable region. ADAM17 is then anchored to the cell via the TMD and cytoplasmic tail. Cleavage site whereby ADAM17 cleaves L-selectin via the metalloproteinase domain is shown by scissors/dotted lines.

L-selectin is a type I transmembrane glycoprotein expressed on leukocytes, involved in leukocyte recruitment to lymph nodes and inflamed tissues. L-selectin function is best characterised on T cells, B cells and neutrophils, with T cells being the focus of this review. L-selectin is encoded by the gene SELL found on human and mouse chromosome 1. The structure of L-selectin is highly conserved between humans and mice, with minor differences in amino acid residues in the cytoplasmic tail and extracellular cleavage site. The molecular weight of L-selectin varies from 65–100 kDa, depending on cell type, due to differences in glycosylation patterns (Figure 3) [98,99].

### 7.1. L-Selectin Ligands and Roles in Lymphocyte Homing

L-selectin was first discovered as a lymph node homing receptor in 1983 using antibody clone MEL-14 against mouse L-selectin. MEL-14 was found to block lymphocyte interactions with HEVs in vitro and reduced lymphocyte recruitment to peripheral lymph nodes in vivo [82]. Further work then characterised vascular addressins such as peripheral lymph node addressin (PNAd), present on peripheral lymph node HEVs using antibody clone MECA-79. These ligands were found to bind to L-selectin on leukocytes and were necessary for cell homing. However, neither MEL-14 nor MECA-79 blocked lymphocyte homing to gut mucosa Peyer’s patches, suggesting a peripheral lymph node-dependent mechanism [83]. 

Further work then discovered that there were other ligands for L-selectin, such as MAdCAM-1 in the mesenteric lymph node of the gut mucosa, and PSGL-1, which binds P- and E-selectin as well [88,100]. Many L-selectin ligands have been characterised since, such as CD34, GlyCAM-1, podocalyxin, nepmucin, endomucin, which collectively make up ‘PNAd’ [101,102,103]. L-selectin binds to the glycan ligand 6 sulpho-sLe^x^ on O- and N-linked sugars presented by the protein backbones of these ligands.

L-selectin is able to control leukocyte homing in the absence of P- or E-selectin [104]. However, mice lacking L-selectin have limited lymphocyte numbers in peripheral lymph nodes, and lymphocytes fail to bind HEVs. Early homing experiments in mice discovered lymphocyte homing to mucosal lymph nodes and Peyer’s patches were all reduced in the absence of L-selectin, further signifying the importance of this molecule [105].

### 7.2. L-Selectin in IAV Infection

Due to its involvement in lymphoid organ homing, a question to address was whether L-selectin is important for homing to tissues during pathology. L-selectin knockout mice displayed reduced lymphocyte, neutrophil and monocyte recruitment to sites of inflammation, whilst early infection studies first implicated L-selectin in the immune response by finding that L-selectin knockout mice had impaired neutrophil responses to peritoneal bacterial infection [105,106]. However, a lack of L-selectin expression on activated CTLs suggested that L-selectin was not important in trafficking T cells to sites of infection [107]. In more recent times, Ager and colleagues overturned the dogma that L-selectin is solely a lymph node homing receptor for lymphocytes and a marker of T cell memory; L-selectin was found to have diverse roles in homing to sites of infection, cancer and in T cell proliferation [2,108,109]. Ager and colleagues also defined an in vivo cyclical expression pattern for L-selectin on CD8^+^ T cells during IAV infection [2].

The cyclical expression of L-selectin was firstly confirmed in vitro, whereby CD8^+^ T cells activated by cognate peptide on APCs completely downregulated L-selectin within 4 h via proteolytic shedding, with re-expression within 48 h due to increased L-selectin mRNA expression. 5–7 days later, L-selectin was downregulated again, by a transcriptional silencing-mediated mechanism controlled by phosphoinositide3-kinase-δ (PI3Kδ) [110,111].

**Figure 4:**
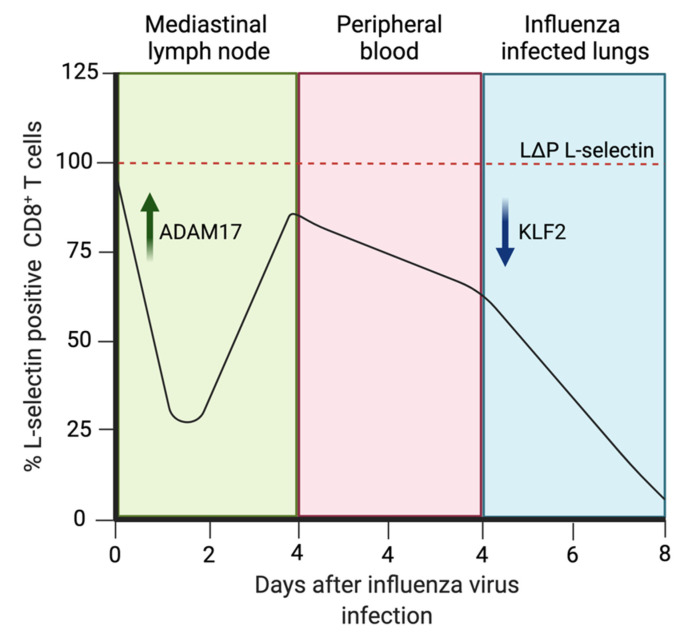
Cyclical expression patterns of L-selectin on CD8^+^ T cells during murine influenza virus infection in vivo. Black line is the L-selectin (CD62L) expression pattern in wild-type mice. Red dashed line is L-selectin expression pattern in LΔP mice, which contain a non-cleavable form of L-selectin that does not undergo transcriptional silencing [2]. The first downregulation of L-selectin is controlled by A disintegrin and metalloproteinase 17 (ADAM17) proteolytic shedding inside lymph nodes. The second downregulation is transcriptional silencing of Kruppel-like factor 2 (KLF2) inside infected tissues [111].

Mohammed et al. used lymphocyte homing experiments in mice to determine the role of L-selectin in CD8^+^ T cells during IAV infection. Naïve CD8^+^ T cells, which express high levels of L-selectin traffic to the mediastinal lymph node via the leukocyte adhesion cascade, is described above. L-selectin on lymphocytes binds to PNAd on HEVs, which directs the cells to the T cell zone of the lymph node, where over 90% of CD8^+^ T cells retain their L-selectin expression for immunosurveillance. Following viral infection with H1N1 A/PuertoRico/8/34 (PR8), these naïve murine T cells become activated into CTLs by APCs exhibiting viral peptide on MHC-I. This interaction between MHC-I/peptide and the TCR results in the proteolytic cleavage of L-selectin on T cells, where less than 40% of antigen-specific CD8^+^ T cells are L-selectin positive. This mechanism is controlled by ADAM17 ectodomain shedding, a PI3Kδ-mediated process. L-selectin levels begin to increase over the following 48 h, when the T cells egress the lymph nodes to reach the systemic circulation. By day 4 of infection, roughly 80% of CTLs express L-selectin in the mediastinal lymph node and 60% in peripheral blood and lungs. L-selectin is downregulated again, being at its lowest in infected tissues by day 8 of infection, where fewer than 5% of antigen-specific CTLs are L-selectin positive. This secondary reduction is controlled by PI3Kδ-mediated transcriptional silencing. PI3Kδ blocks the action of the transcription factor Kruppel-like factor 2 (KLF2), which transcribes the L-selectin gene SELL [2,99,111]. This identifies an in vivo cyclical expression pattern for L-selectin on CD8^+^ T cells, which supports previous in vitro findings [110] (Figure 4).

Antibody MEL-14 was historically used to identify L-selectin on leukocytes and has been successful at blocking the functionality of this molecule. During PR8 influenza virus infection, daily administration of MEL-14 to mice after T cells had been activated inside lymph nodes significantly reduced numbers of influenza-specific CD8^+^ T cells in the lungs on day 8 of infection, suggesting an L-selectin-dependent mechanism for CTL trafficking to sites of infection [2].

To further explore the functional role of L-selectin expression on activated T cells, genetically modified ‘LΔP’ mice were generated, in which ADAM17-resistant L-selectin is expressed on the T cells under a heterologous promoter [112]. Using these LΔP mice, maintained L-selectin expression on CTLs was found to improve CD8^+^ T cell homing and subsequent IAV clearance on day 8 in both H1N1 and H3N2 infections [2]. This markedly improved virus clearance in LΔP mice compared to wild-type C57BL/6 (B6) mice was further emphasised by poorer virus clearance in L-selectin^−/−^ knockout mice compared to wild-type [2].

These findings open up a potential avenue of targeting L-selectin pharmacologically to treat IAV or other infections; however, a mechanism of action is required to fully understand the downstream effects. Cell activation and differentiation remained the same between LΔP and B6 murine CD8^+^ T cells, suggesting that the superior homing effects are not due to early or increased cell activation [2]. This proposes a cell migration-mediated superiority in LΔP CTLs, in which expression of ligands for L-selectin on endothelium would be key. Traditional ligands for L-selectin; PNAd and MAdCAM-1 are not detectable in murine influenza-infected lungs (unpublished data, Ager lab), suggesting that other ligands not conventionally found in HEVs or vascular endothelium are responsible for this mechanism.

## 8. ADAMs

ADAMs are a family of type I transmembrane enzymes or ‘sheddases’, which function to cleave the ectodomains of other transmembrane molecules. Ectodomain shedding is a proteolytic reaction, in which an extracellular domain is removed, leaving a membrane-retained fragment in place. To date, 21 ADAMs have been characterised in humans, with 13 being proteolytically active [76]. The proteolytically active ADAMs have an active site attached to the metalloproteinase domain for ectodomain shedding (Figure 4) [76,113]. A full-length precursor and a mature form of ADAM proteins are detected in cells; ADAMs are synthesised in the endoplasmic reticulum (ER) and matured in the Golgi. The maturation process involves glycosylation and removal of the pro-domain via proprotein convertase 7 or furin, to create a functional metalloproteinase domain. ADAMs are found to predominantly localise to the perinuclear and plasma membranes in order to cleave substrates in cis [114].

ADAM17 was the first of the ADAMs to be described. It is also known as tumour necrosis factor alpha converting enzyme (TACE), named after its initial discovery in cleaving transmembrane TNFα into soluble TNFα [115,116]. By using ADAM17 gene knockout mice, the protease was found to be essential for survival, as mice died between embryonic day 17.5 and 1 day post-birth [117]. This lethality was attributed to aberrant epidermal growth factor receptor (EGFR) signalling [117]. 

The signalling pathway for ADAM17-dependent proteolytic shedding of membrane inserted substrates involves stimulation of receptors such as toll like receptors (TLRs), TCRs, GPCRs or pharmacological activation using phorbol esters. This induces mitogen-activated protein-kinase (MAP-kinase)-dependent phosphorylation of the cytoplasmic tail of rhomboid protein iRhom2. MAP-kinase phosphorylation can occur via an array of proteins, such as protein kinase C (PKC) or PI3Kδ, which depends on cell stimuli. iRhom2 transports immature ADAM17 from the ER and allows pro-domain cleavage to release mature ADAM17, which is then trafficked to the cell membrane, also by iRhom2. This process results in ADAM17 ectodomain cleavage of other transmembrane proteins [118,119].

ADAM17 currently has over 80 known substrates including chemokines, cytokines, growth factors and receptors [78]. Its ‘promiscuous’ activity and role in regulating the bioavailability of various molecules has implicated ADAM17 in cancer, neurological and inflammatory immune conditions, as well as leukocyte recruitment. 

### 8.1. ADAM17 in Immunity

ADAM17 has functions in regulating many cell surface receptors and molecules which influence the immune response to infection (Table 3). This includes pattern recognition receptors (PRRs) TLR2 and TLR4; other first line defenses that detect foreign pathogens during an infection, as well as a number of inflammatory mediators. TNFα is a pro-inflammatory cytokine central to orchestrating immune responses but can also cause pathology through excessive inflammation. TNFα binds to TNFRI and TNFRII to elicit its inflammatory pathway. Inhibition of ADAM17-mediated cleavage of TNFα on CD8^+^ T cells improves survival in murine influenza infection with reduced lung pathology. This was attributed to reduced CXCL2 expression in mouse lungs and therefore reduced inflammatory innate immune cell infiltration [120]. Soluble TNFRII, another substrate of ADAM17 released by CD8^+^ T cells, was also shown to regulate soluble TNFα levels and therefore regulate inflammation during IAV infection [121].

The processes of leukocyte migration and recruitment also involve ADAM17, via cleavage of chemokines and cell adhesion molecules such as ICAM-1, VCAM-1 and L-selectin [134,135,138]. These molecules have pronounced effects on the cell adhesion cascade which takes place in blood vessels and HEVs, where leukocyte rolling, arrest and transendothelial migration result in leukocyte recruitment to lymph nodes and tissues. This is important for both homeostatic immune surveillance, and during infection, where immune cells traffic to sites of inflammation [76].

#### 8.1.1. ADAM17 Regulation of L-Selectin Expression by T cells

ADAM17 was discovered to cleave the ectodomain of the transmembrane homing molecule L-selectin in 1998 [117]. ADAM17 cleaves L-selectin at a single site in the 15 amino acid extracellular membrane proximal region. This cleavage is mediated by the metalloproteinase domain of ADAM17 [76]. The hypervariable region of the cysteine-rich domain in ADAM17 could also regulate L-selectin shedding [76]. ADAMs 8 and 10 have also been found to cleave L-selectin under certain conditions, however, ADAM17 is the dominant enzyme involved (Figure 3), [142,143]. On T cells, TCR stimulation/cross-linking induces PKC activation, which initiates the PI3Kδ pathway and subsequent rapid ADAM17-mediated shedding [110,111,144]. In vitro, L-selectin begins to downregulate from T cells from 1-h post-activation, with 90% shed by 4 h [110].

Mohammed et al. found that constitutive shedding of L-selectin on naïve leukocytes is not ADAM17- or ADAM10-mediated; this process is still not fully elucidated. However, shedding of L-selectin on peptide-MHC activated CD8^+^ T cells was found to be ADAM17-dependent and a fundamental mediator of cell proliferation. In vitro murine CD8^+^ T cells expressing a shedding-resistant form of L-selectin showed reduced proliferation and CD25 expression compared to wild-type CD8^+^ T cells, when stimulated by cognate peptide. These results were also seen in mice infected with vaccinia virus in vivo, whereby wild-type CD8^+^ T cells clonally expanded 8-times more than CD8^+^ T cells expressing a shedding-resistant mutant of L-selectin [109]. These results demonstrate the functional relevance of ADAM17-mediated cleavage of L-selectin at sites of immune activity and illustrate non-homing related effects which have implications for T cell function. Although the maintenance of L-selectin on CD8^+^ T cells is shown to improve homing to sites of virus infection and subsequent viral clearance, lack of proteolytic shedding may reduce early lymphocyte proliferation and impede the initial adaptive immune response to infection [2,109]. 

#### 8.1.2. ADAM17 in Infection

Due to its diverse array of substrates, it is no surprise that ADAM17 plays an important role in controlling infection. Impairing ADAM17 function in a murine model of bacterial peritonitis enhanced both L-selectin and TNFα cell-surface expression on neutrophils which increased neutrophil recruitment to the peritoneum, reduced bacterial load and improved survival [145]. Neutrophils expressing a non-cleavable form L-selectin alone were found to confer an advantage in early neutrophil recruitment in bacterial peritonitis, but not in bacterial clearance. This suggests that substrates of ADAM17 other than L-selectin may contribute to this protection [146]. Conversely, ADAM17 dependent shedding of L-selectin is required for neutrophils to clear Klebsiella pneuomiae infection from the lungs of mice [147]. This suggests different requirements for L-selectin shedding depending on leukocyte type, pathogen and target tissue.

ADAM17 was found to contribute to lung pathology in murine IAV infection via cleavage of CD8^+^ T cell TNFα which induced CXCL2 expression on lung epithelium, leading to heightened inflammatory cell infiltration. Inhibition of CD8^+^ T cell ADAM17 in this mouse model reduced lung injury and improved survival [120]. ADAM17 cleavage of TNFRII on CD8^+^ T cells was also found to reduce bioavailability of circulating soluble TNFα in IAV infection [121].

L-selectin is distinctively known not just as a marker of leukocyte homing, but also a marker of central memory T cells (T_CM_). In humans, memory T cells that persist after infection to prevent recurring disease, can be broadly divided into CCR7^HI^CD62L^HI^ central T_CM_ that survey secondary lymphoid organs, and CCR7^LO^CD62L^LO^ effector memory T cells (T_EM_) that survey tissues. There is evidence to suggest that ADAM17-controlled L-selectin shedding is required for effective CD8^+^ T cell memory responses to recurrent virus infection in mice. Mice containing non-shedding LΔP L-selectin on lymphocytes conferred delayed protection against secondary virus challenge. Influenza-specific CD8^+^ memory T cell quantity and distribution were similar between wild-type and LΔP mice, apart from within lymph nodes where LΔP mice contained more cells. Upon secondary challenge, the recruitment of CD8^+^ memory T cells to the site of infection was comparable between groups, as well as cell differentiation, the only other difference observed bar viral clearance kinetics, was that LΔP memory CD8^+^ T cells expressed higher IFNγ and TNFα levels [148]. These results are contrasting to findings in primary IAV infection, suggesting L-selectin plays different roles in CTLs vs memory CD8^+^ T cells in virus infection.

#### 8.1.3. Pharmaceutical Agents to Block ADAM17

Due to indication of roles for ADAM17 in several diseases, it has become a pharmaceutical target. Small molecule inhibitors have been developed, such as dual ADAM10 and ADAM17 inhibitor INCB7839/Aderbasib, which is currently being tested in clinical trials for paediatric glioma as well as diffuse large B cell lymphoma (DLBCL) in combination with rituximab, post autologous haematopoietic cell transplant [149,150]. Other small molecule inhibitors against ADAM17 such as BMS 561392/DPC-333 and Apratastat/TMI-005 are also available; however, these are known to have off-target effects on other matrix metalloproteinases (MMPs) and failed to progress through clinical trials due to toxicity and efficacy concerns [151,152].

Further efforts to find specific inhibitors of ADAM17 have focused on developing monoclonal antibodies. Short-chain variable fragment (scFv) clones against ADAM17 epitopes have been identified using phage display technology, then developed into full length antibodies. D1(A12) is a human anti-ADAM17 ‘cross-domain’ IgG antibody, which contains the variable heavy (V_H_) chain of a human IgG antibody that binds a non-catalytic domain of ADAM17, and the variable light (V_L_) chain that binds to the catalytic domain of ADAM17 [153]. A9(B8) is a mouse and human cross-reactive anti-ADAM17 antibody, formatted onto a human IgG2 framework [154]. MEDI3622 is another mouse and human cross-reactive anti-ADAM17 antibody, on a IgG1 framework [155]. Each of these antibodies has been shown to be efficacious in a range of indications both in vitro and in vivo but are yet to be tested in human trials (Table 4).

Toxicity and efficacy issues with ADAM17 inhibitors led to discontinuation of human trials. This may not be a surprising finding since patients with mutations in ADAM17 being documented to have serious clinical conditions. Twins identified to have a loss-of-function mutation of ADAM17 suffered severe skin infections, bowel lesions and cardiomyopathy, with one twin dying at age 12 from myocarditis [178]. Another individual was identified with a frameshift mutation in ADAM17, leading to similar symptoms and death at 10 months due to recurrent sepsis [179].

## 9. Conclusions

IAV is a clinically relevant virus which circulates yearly, causing significant morbidity and mortality worldwide. The immune response to IAV infection requires a multitude of cells from both the innate and adaptive immune system to orchestrate protection to the host. Leukocyte homing to lymph nodes and tissues is fundamental for immunosurveillance and response to pathogens. This underpins the clearance of IAV, as cells must traffic to the correct tissues and organs to effectively eradicate the infection. Several selectins, chemokine receptors, integrins and their ligands co-ordinate this migration, with L-selectin being one of the most researched and the first homing receptor linked to directly improving virus clearance.

**Figure 5:**
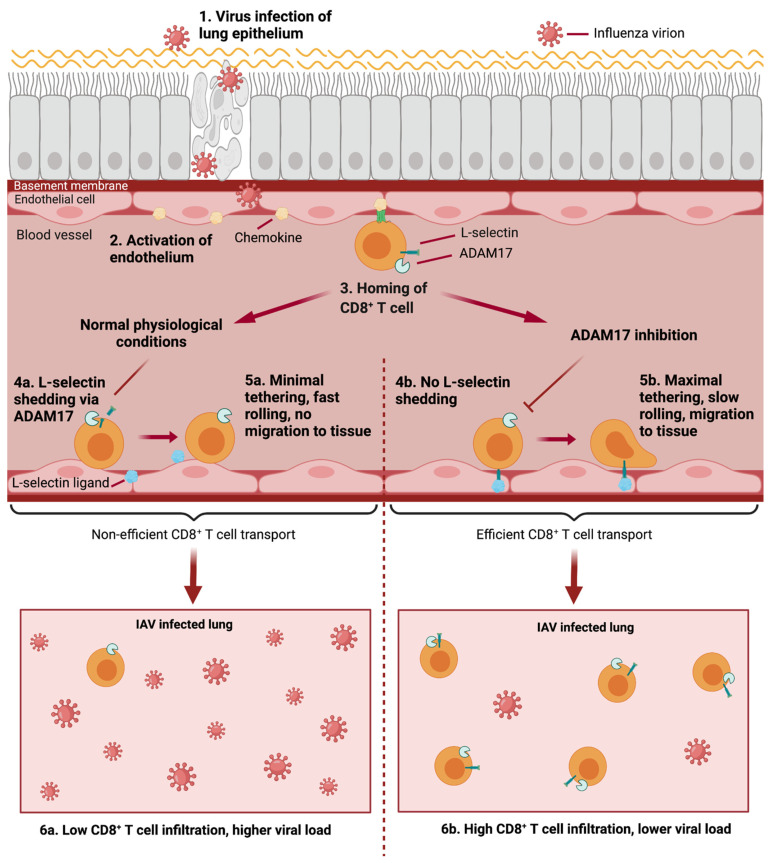
The role of ADAM17-mediated control of L-selectin in CD8^+^ T cell homing in IAV infection. **1.** Influenza virions infect airway epithelium. **2.** Epithelial infection results in activation of blood vessel endothelial cells and expression of chemokines. **3.** During homing, CD8^+^ T cells become activated by chemokines, stimulating A disintegrin and metalloproteinase 17 (ADAM17). This leads to two possible outcomes depending on the level of ADAM17-dependent shedding of L-selectin (CD62L). Under normal conditions: **4a.** L-selectin will become proteolytically cleaved by ADAM17. **5a.** Loss of L-selectin ectodomain results in minimal CD8^+^ T cell tethering and rolling along endothelium. T cells fail to migrate to influenza A virus (IAV) infected tissue. **6a.** IAV infected lungs will contain lower CD8^+^ T cell infiltration and will therefore result in higher viral load. When ADAM17 dependent L-selectin shedding is inhibited: **4b.** Blocking ADAM17 function retains L-selectin expression on the surface of CD8^+^ T cells. **5b.** L-selectin binding to its ligand allows maximal CD8^+^ T cell tethering and rolling along endothelium. T cells migrate to influenza A virus (IAV) infected tissue. **6b.** IAV infected lungs will contain higher CD8^+^ T cell infiltration and will therefore result in lower viral load [2].

L-selectin and ADAM17 have clear fundamental roles in controlling the migration of leukocytes during infection [2,105,120,145] (Figure 5). However, the mechanisms behind the protective effects seen by blocking L-selectin shedding and/or ADAM17 function are still to be fully elucidated. New unidentified ligands for L-selectin are prime candidates for this mechanism; these ligands may be present in inflamed HEV, peripheral blood vessels or in infected tissues and may help guide leukocytes to infected organs to elicit cytotoxic responses. There is yet to be clear understanding of the regulation of cell surface L-selectin by ADAM17. Understanding of mature ADAM17 cell surface expression patterns alongside transcription and translation of the L-selectin gene SELL are required in vivo. The translatability of murine data must also be tested, as there are limited primary human data available on functional L-selectin expression on leukocytes. Primary human data available suggest L-selectin may have crucial roles in T_EM_ homing, as well as in T cell responses during HIV infection [180,181]. 

There are several approaches being used to pharmacologically inhibit ADAM17 in various diseases from infection to autoimmune diseases and cancer. A major downfall with targeting ADAM17 is the off-target effects resulting from the promiscuity of this protein; many substrates will be impacted, which may have undesired effects on patients. Other therapeutic avenues must be considered such as genetic modification and infusion of autologous T cells or drugs that selectively target the ADAM17 cleavage site of L-selectin.

## Figures and Tables

**Table 1:** Innate leukocytes recruited to the lungs during murine influenza A virus (IAV) infection and their roles in immunoprotection and immunopathology. Leukocytes with crucial roles in IAV infection are listed, along with the timepoints of maximal accumulation in the lung. Roles in immunoprotection and immunopathology to the host in IAV infection are listed. IFN; interferon, NETosis; neutrophil extracellular trap(osis), CTL; CD8^+^ cytotoxic T lymphocyte, ROS; reactive oxygen species.

Leukocyte	Peak Accumulation in the Lung Following IAV Infection	Immunoprotection Roles	Immunopathology Roles
Monocyte	5 days [36]	Pro-inflammatory cytokine production [37], differentiation into dendritic cells [9]	Excessive pulmonary inflammation and tissue damage leading to mortality [38]
Alveolar macrophage	2 days [39]	Phagocytosis of infected cells [39], prevention of epithelial cell infection [40]	Excessive pulmonary inflammation and tissue damage leading to mortality [41]
Dendritic cell	10 days [36]	Type I IFN production [42], activation and recruitment of antigen-specific T cells [36]	Excessive pulmonary inflammation and tissue damage leading to mortality [43]
Neutrophil	3–5 days [44]	Pro-inflammatory cytokine production [44], phagocytosis of infected cells [39], anti-bacterial granule release and NETosis to limit viral replication [45], recruitment of CD8^+^ T cells [46]	Excessive pulmonary inflammation, ROS production [47] and tissue damage leading to mortality [48]
Natural killer cell	2–4 days [49], 6 days [50]	Pro-inflammatory cytokine production [51], cytotoxicity of IAV infected cells [49], recruitment of dendritic cells and T cells to mediastinal lymph node [52], enhancement of CTL responses [53]	Excessive lung inflammation, increased phagocyte and neutrophil recruitment [54]

**Table 2:** Adhesion molecules required for T lymphocyte homing. Lymphocyte homing to lymph nodes, Peyer’s patches in the gut and lungs in IAV infection require a unique set of adhesion molecules that aid in tethering, rolling and arrest of lymphocytes for cell trafficking. PNAd; peripheral lymph node addressin, CCR7; chemokine receptor 7, CCL19/21; chemokine ligand 19/21, LFA-1; leukocyte function-associated antigen 1, ICAM-1; intercellular cell-adhesion molecule-1, MAdCAM-1; mucosal addressin cell adhesion molecule 1, PSGL-1; P-selectin glycoprotein ligand 1, IAV; influenza A virus, VLA-4; very late antigen-4, Mac-1; macrophage antigen-1, VCAM-1; vascular cell-adhesion molecule-1.

Location	Selectins	Selectin Ligands	Chemokine Receptors and Ligands	Integrins	Integrin Ligands
Peripheral lymph nodes	L-selectin [82]	PNAd [83]	CCR7, CCL19, CCL21 [57,84,85]	LFA-1 [86]	ICAM-1, ICAM-2 [86,87]
Mesenteric lymph node	L-selectin [82]	MAdCAM-1, PNAd [83,88]	CCR7, CCL19, CCL21 [89]	α4β7, LFA-1 [90,91]	MAdCAM-1 [90]
Peyer’s patches in gut mucosa	P-selectin ^1^, L-selectin [92]	PSGL-1, MAdCAM-1 [93]	CCR7, CCL21 [94]	α4β7, LFA-1 [91,95]	MAdCAM-1 [95]
Lungs in IAV infection ^1^	L-selectin [2]	Not known	CXCR1, CXCR3, CCR4, CCR5, CXCR6 [96]	VLA-4, LFA-1 [96]	VCAM-1, ICAM-1 [96]

^1^ Required during inflammation/infection.

**Table 3:** Substrates of ADAM17 in humans and mice and their functions within the immune system. TLR; Toll like receptor, IL-6/15/23R; interleukin 6/15/23 receptor, IL-1RII; interleukin 1 receptor II, TNFR-I/II; tumour necrosis factor receptor I/II, TNFα; tumour necrosis factor alpha, IL-1β; interleukin 1 beta, CD154/89/44/16; cluster of differentiation 154/89/44/16, ICAM-1; intercellular adhesion molecule 1, VCAM-1; vascular adhesion molecule 1, JAM-A; junction adhesion molecule A, EGF; epidermal growth factor, LAG-3, lymphocyte activation gene 3, TIM-3/1/4; T cell immunoglobulin and mucin domain 3/1/4, ACE2; angiotensin converting enzyme 2.

Role within the Immune System	ADAM17 Substrate
Pattern recognition	TLR2 [122], TLR4 [123]
Inflammation	IL-6R [124], IL-15R [125], IL-23R [126], IL-1RII [127], TNFR-I [117], TNFR-II [128], TNFα [115], Lymphotoxin-αβ [129], IL-1β, [130], CD154 [131], CD89 [132], 4-1BB [133]
Leukocyte adhesion and migration	ICAM-1 [134], VCAM-1 [135], L-selectin [117], CD44 [136], JAM-A [137]
T cell activation, proliferation and exhaustion	LAG-3 [119], TIM-3 [138]
Natural killer cell toxicity	CD16 [139]
Viral cell entry	ACE2 [140], TIM-1, TIM-4 [141]

**Table 4:** ADAM17 inhibitors and disease models tested. A disintegrin and metalloproteinase 17 (ADAM17) inhibitor drugs, their formulations and published research into treatment of various disease models in vitro and in vivo using mouse models or human clinical trials. scFv; small chain variable fragment, NSCLC; non-small cell lung cancer, HNSCC; head and neck squamous cell carcinoma, DLBCL; diffuse large B cell lymphoma, HCT; haematopoietic cell transplant.

Drug Name	Formulation	In Vitro Models	Mouse Models	Human Trials
INCB7839/ Aderbasib	Small molecule inhibitor	HER2 + breast cancer with trastuzumab [156]	High-grade glioma xenograft tumour model [157]	Phase I; paediatric glioma [150]
				Phase I/II; DLBCL in combination with rituximab, post autologous HCT [149]
			HER2+ breast cancer xenograft tumour model with trastuzumab [156]	Phase I; HER2+ metastatic breast cancer with trastuzumab [158]
BMS-561392/ DPC-333	Small molecule inhibitor	NSCLC [159]	Collagen-induced arthritis (CIA) model [161]	Phase II rheumatoid arthritis [161]
			Autoimmune hepatitis [162]	
		Alzheimer’s [160]	Acute colitis [163]	
			Alzheimer’s model [160]	
			Spinal cord injury [164]	
TMI-005/ Apratastat	Small molecule inhibitor	Polycystic kidney disease [165].	SARS-CoV-2 [166]	Phase II clinical trial for rheumatoid arthritis [152].
			NSCLC [167].	
JTP-96193	Small molecule inhibitor		Type 2 diabetes & diabetic peripheral neuropathy [168].	
D1(A12)	Human IgG antibody	HNSCC [169].	Ovarian xenograft tumour model [170].	
		Triple negative breast cancer [171]		
A9(B8)	Humanised mouse IgG2 antibody	NSCLC with erlotinib/ gefitinib [172].	Pancreatic cancer [173].	
MEDI3622	Humanised mouse IgG1 antibody	Colorectal cancer [174].	Head and neck & colorectal xenograft tumour models [175]	
		Ovarian cancer & Burkitt’s lymphoma [176]	Polymicrobial sepsis [177].	
